# New Publications

**Published:** 1876-04

**Authors:** 


### New Publications.

Messrs. J. H. Coates & Co., 822 Chestnnt St., Philadelphia, Pa., announce a new monthly to be conducted by Carl Seiler, M. D., in conjunction with J. G. Hunt, M. D. and Joseph G. Richardson, M. D. The title of the publication will be Micro-Photographs in Histology, Normal and Pathological.

The object of this work is to replace, as far as possible, the microscope for those physicians who have neither the time nor opportunity to make observations with the instrument for themselves, and also to furnish microscopists for comparison, correct representations of typical specimens in domain of normal and pathological histology. Each number will contain at least four plates with descriptive letter press. The numbers will be sold at sixty cents each, or at $6.00 per volume of twelve numbers.——The Archives of Clinical Surgery is announced shortly to appear in New York. Several of the best lecturers upon Surgery in New York and elsewhere will contribute to its pages.

